# Cardiac Differentiation Promotes Focal Adhesions Assembly through Vinculin Recruitment

**DOI:** 10.3390/ijms24032444

**Published:** 2023-01-26

**Authors:** Flavia Carton, Simona Casarella, Dalila Di Francesco, Emma Zanella, Annarita D’urso, Luca Di Nunno, Luca Fusaro, Diego Cotella, Maria Prat, Antonia Follenzi, Francesca Boccafoschi

**Affiliations:** 1 Department of Health Sciences, University of Piemonte Orientale “A. Avogadro”, 28100 Novara, Italy; 2 Tissuegraft Srl, 28100 Novara, Italy

**Keywords:** cardiac differentiation, focal adhesions, vinculin, FAK, mechanical stress, mechanotransduction

## Abstract

Cells of the cardiovascular system are physiologically exposed to a variety of mechanical forces fundamental for both cardiac development and functions. In this context, forces generated by actomyosin networks and those transmitted through focal adhesion (FA) complexes represent the key regulators of cellular behaviors in terms of cytoskeleton dynamism, cell adhesion, migration, differentiation, and tissue organization. In this study, we investigated the involvement of FAs on cardiomyocyte differentiation. In particular, vinculin and focal adhesion kinase (FAK) family, which are known to be involved in cardiac differentiation, were studied. Results revealed that differentiation conditions induce an upregulation of both FAK-Tyr397 and vinculin, resulting also in the translocation to the cell membrane. Moreover, the role of mechanical stress in contractile phenotype expression was investigated by applying a uniaxial mechanical stretching (5% substrate deformation, 1 Hz frequency). Morphological evaluation revealed that the cell shape showed a spindle shape and reoriented following the stretching direction. Substrate deformation resulted also in modification of the length and the number of vinculin-positive FAs. We can, therefore, suggest that mechanotransductive pathways, activated through FAs, are highly involved in cardiomyocyte differentiation, thus confirming their role during cytoskeleton rearrangement and cardiac myofilament maturation.

## 1. Introduction

Focal adhesions (FAs) are complex multi-protein structures that mediate cell adhesion by connecting cytoskeleton to the extracellular matrix (ECM) and contribute to the cell migration by translating external forces on the actin stress fibers. Depending on their number, size, and composition, in combination with myosin-II contractility, FAs transmit and transduce mechanical force from the cytoskeleton to the ECM, acting as mechanosensors [1,2]. The ability of cells to convert mechanical cues, sourced from the surrounding environment, into intracellular biochemical signals is of primary importance in a mechanically active tissue, such as myocardium, in which coordinated muscle contraction depends on proper mechanical and electrical coupling [3]. Within the cardiac tissue, mechanical forces are transmitted to the actomyosin through a specialized FA complex known as the costamere [4].

FA nanoarchitecture shows actin stress fibers linked to the integrin transmembrane receptors through FA plaque proteins. In detail, during FA formation, the binding of integrin transmembrane receptors with ECM proteins induces conformational changes and clustering of integrins. This process is followed by the recruitment of different intracellular early adaptor proteins (e.g., talin and paxillin) that, in turn, promote the clustering and activation of further integrins. Secondary, post-translationally modified proteins bind the early adaptor, activating different signaling pathways and creating new binding sites. Finally, actin stress fibers directly involved in cytoskeletal remodeling are recruited [5,6,7].

Once definitive FAs are formed, three different layers can be identified: the bottom layer, closest to the cell membrane, the middle layer, responsible for the transmission of force, and the top layer, directly associated with the actin stress fibers [8]. In the bottom layer, one of the first proteins that paxillin recruits is FAK, a non-receptor tyrosine kinase, which auto-phosphorylates in Tyr397 and, in turn, phosphorylates Src and paxillin in a mechanosensitive way, influencing FA size.

The middle layer consists of talin and vinculin proteins. Among them, vinculin plays a pivotal role in regulating FA formation and turnover, anchoring the sarcomere to the cell membrane [2,9,10,11]. Structurally, vinculin consists of an N-terminal head, a proline-rich region (neck), and a C-terminal tail domain [12]. Conformational changes in these domains drive vinculin activation and consequently FA formation. Indeed, in the extended form (active state), vinculin interacts with talin, having a direct effect on integrin clustering and consequently on FA maturation [9,13,14]. Moreover, the confinement of vinculin in FAs determines the recruitment of paxillin, which in turn promotes the recruitment and interaction of other proteins involved in FA formation. However, other mechanisms are involved in vinculin activation. For instance, during FA development, residues Y100 and Y1065 are phosphorylated, enhancing the activation of vinculin as well as the binding of PIP2 to vinculin tail [2,15,16,17,18]. Lastly, vinculin can cross-link and bundle actin filaments and also modify the existing ones, stimulating the formation of new bundles through its different binding sites for VASP, Arp2/3 complex (an actin nucleator) [13,14].

All this evidence confirms the important role of vinculin in actin cytoskeletal dynamics and, thus, not only in maintaining an adequate functionality in cardiac tissue but also in cardiac morphogenesis. The literature reports that loss of vinculin in myocardium leads to destabilization of adherent junctions, gap junctions and costameres, resulting in an inadequate mechanical and electrical coupling [19,20,21].

Integrins work also with several factors, such as focal adhesion kinase (FAK), which has been proposed to be an important modulator of ECM-associated signaling pathways. In particular, FAK auto-phosphorylates and creates a binding site for Src-family proteins and Cas that become phosphorylated. FAK-Cas, in combination with multimolecular complexes, promotes the activation of GTPases of Ras and Rho families, leading to the downstream stimulation of MAPK cascades that regulate cytoskeletal reorganization and differentiation [1,16,17,18]. Interestingly, these kinases have been shown to be also directly or indirectly linked to the Wnt signaling pathway via regulation of GSK3β, another critical point for cardiomyogenesis [22,23,24].

Moreover, FAs are also the main regulators of tension-dependent cellular changes [25,26,27,28,29,30]. Mechanical forces are essential to regulate cardiac differentiation during heart morphogenesis. Indeed, through cardiac development, myocardial tissue begins to fold and, with the onset of chamber pumping, cardiac progenitors and later cardiomyocytes are subjected to stretching forces [26,27]. Both active and passive forces are mediated by integrin-based cell adhesions [26,27]. A mechanotransductive hypertrophic response includes a rapid induction of immediate-early genes, such as c-fos, c-jun, and c-myc, that act as transcription factors and lead to changes in gene expression, including the transition of sarcomeric proteins from the fetal form to the adult [28,31,32]. The literature suggests that FAK/Src complex mediates hypertrophic growth and survival signaling after a mechanical load, leading to activation of some pathways such as Ras cascade, NF-kB activity, MAPK/ERK signaling and hippo pathway [25,29,32,33]. Furthermore, several studies outlined how integrin expression is upregulated in response to mechanical stimuli, improving cell adhesion, FA assembly and cardiomyogenesis [27,34,35]. Besides, structural analysis has shown that vinculin conformation is usually folded in a latent state but becomes activated when mechanical force is applied [10,36,37]. Thus, in the areas of sustained stress, FAs start to be assembled, resulting in the activation of downstream regulators, such as kinases, phosphatases, and transcriptional modulators, which in turn regulate the actomyosin complex and remodeling of the heart [38]. However, how exactly the ECM–integrin–cytoskeletal complex senses mechanical stimuli still requires further investigations [39].

To provide additional experimental evidence on the regulatory aspect of FAs in cell differentiation and cell mechanical stability, the present work focused on the role of vinculin and FAK in H9c2 cardiomyocytes.

## 2. Results

### 2.1. Increase in Cardiac Markers in Differentiated Cells

**Figure 1:**
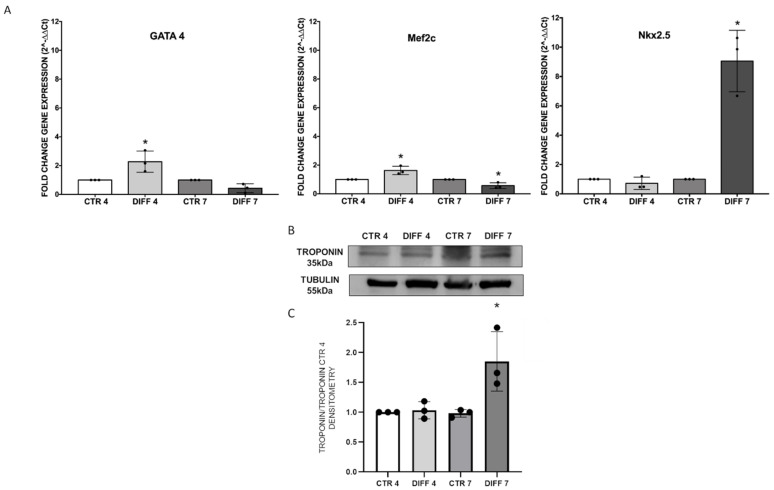
H9c2 myoblast differentiation towards a cardiac-like phenotype after cells were cultured with retinoic acid (RA) for 4 and 7 days. (**A**) The relative gene expression of cardiomyogenic markers GATA 4, Mef2c and Nkx2.5 was assessed by q-RT-PCR. Results were expressed as fold-change ± SD with respect to the untreated cells, obtained in three independent experiments performed in triplicate and analyzed by two-way ANOVA with Bonferroni’s multiple comparison test (* *p* ≤ 0.05). (**B**) Western blot analysis with mouse anti-cardiac troponin T (cTnT) and anti-tubulin antibodies on H9c2 cell lysates after 4 and 7 days of differentiation. Proteins were revealed on total protein content, and all results were normalized with respect to control at day 4. (**C**) Densitometry results obtained from three different experiments are expressed as mean ± SD. Statistical significance with respect to the relative controls (* *p* ≤ 0.05).

Cardiomyogenic differentiation of H9c2 cells was examined through gene expression of some cardiomyogenic commitment markers: GATA-4, Mef2c and Nkx2.5. Results showed that GATA-4 and Mef2c, two early cardiac markers expressed at the beginning of differentiation, were upregulated after 4 days with respect to the control. On the contrary, the cardiac transcription factor Nkx2.5, was significantly expressed by day 7 of differentiation (Figure 1A). To further confirm cardiomyoblast differentiation, the expression of cardiac troponin T (cTnT), a protein involved in the functional maturation of cardiomyocytes, was evaluated by Western blot, showing a significant upregulation at day 7 with respect to controls (Figure 1B,C). The results obtained confirm the proper differentiation of H9c2 cardiomyoblasts.

### 2.2. Activation of FAK and Vinculin Translocation during Cardiomyocyte Differentiation

In view of the role played by FAs during cardiomyogenesis, the presence, localization and activation of some adhesion proteins were studied.

**Figure 2:**
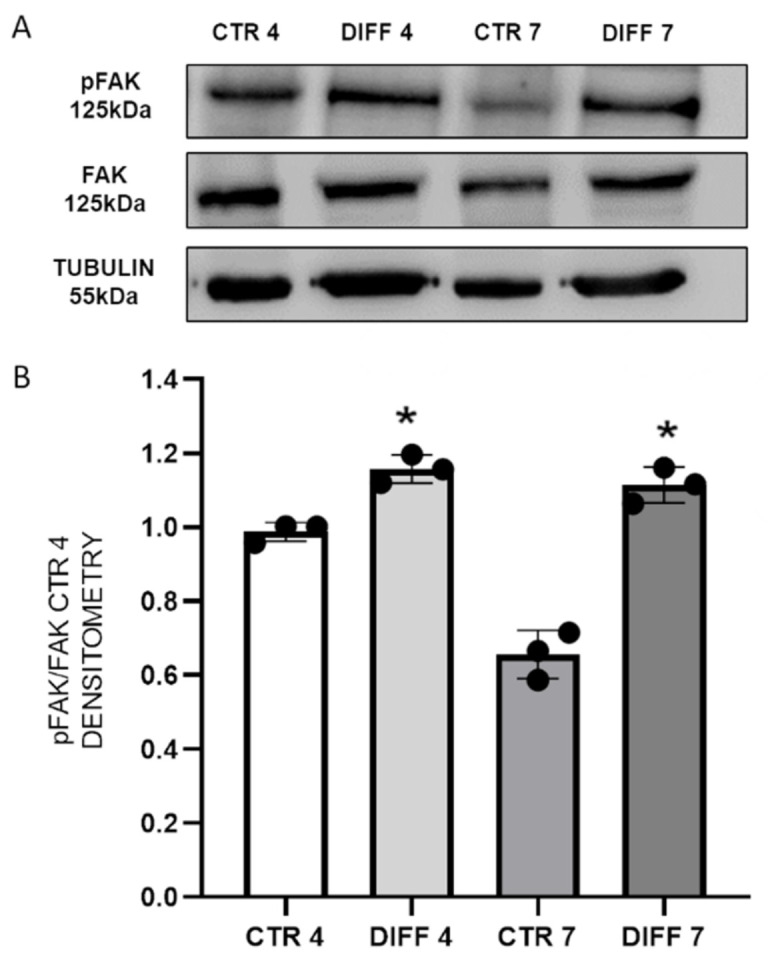
(**A**) Western blot analyses with anti-pFAK, anti-FAK and anti-tubulin antibodies on H9c2 cell lysates after 4 and 7 days of differentiation. Proteins were revealed on total lysates, and all results were normalized with respect to FAK expression at day 4. (**B**) Densitometry results obtained from three different experiments are expressed as mean ± SD. Statistical significance with respect to relative controls (* *p* ≤ 0.05).

FAK activation was evaluated by Western blot. This kinase was selected because it is a primary integrin effector that acts as a node for the convergence of multiple signaling pathways involved in cardiomyogenesis. FAK activation is ensured by the phosphorylation of tyrosine residue Tyr-397, which recruits Src family kinases that in turn promote other signaling pathways involved in cardiac differentiation. Figure 2 shows a significant increase in pFAK at 4 and 7 days in differentiated cells with respect to controls.

**Figure 3:**
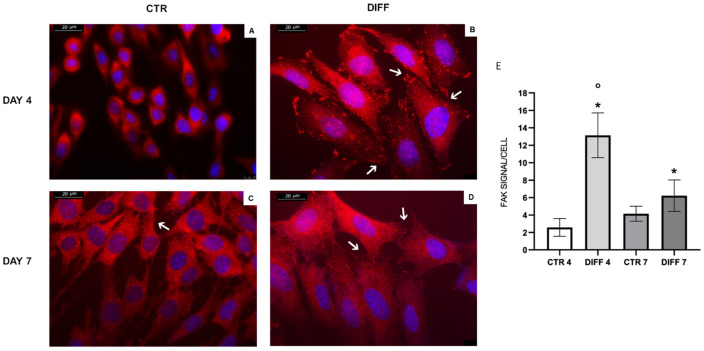
FAK (red) staining in H9c2 cells cultured for 4 and 7 days in growth (**A**–**C**) and differentiated medium (**B,D**). Nuclei were stained with DAPI (blue). Images are representative of all of the results obtained in the different experimental conditions. White arrows indicate FAK signal in focal adhesions. Scale bar: 20 µm. (**E**) The number of actin-related FAK-positive signals in each condition is presented in the graph. Statistical significance with respect to the relative controls (* *p* ≤ 0.05); statistical significance with respect to differentiated cells at day 4 (° *p* ≤ 0.05).

To provide further insights on FAK activation, anti-FAK Ab staining was performed (Figure 3). Results showed that a ubiquitous signal in the cytoplasm was detected in the controls, and actin-related FAK signals became significantly more evident in differentiated cells, particularly at day 4, as shown in Figure 3E.

**Figure 4:**
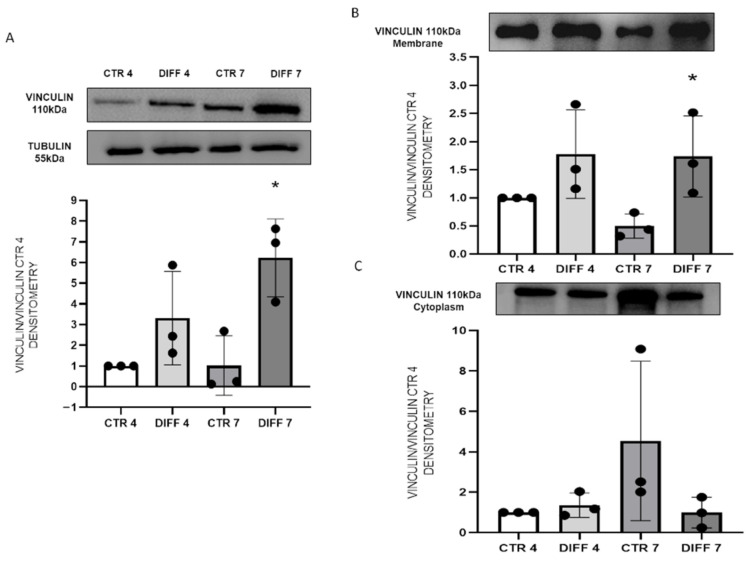
Western blot analyses with anti-vinculin and anti-tubulin antibodies on H9c2 after 4 and 7 days of differentiation. Proteins were revealed (**A**) on total proteins, (**B**) on membrane fraction, and (**C**) on cytoplasm fraction. All results were normalized with control at day 4. Densitometry results obtained from three different experiments are expressed as mean ± SD. Statistical significance with respect to relative controls (* *p* ≤ 0.05).

In addition, FA formation and H9c2 differentiation were also investigated through the detection of vinculin, one of the major components of FAs. Vinculin expression and membrane recruitment were performed by Western blot analysis both on total proteins and subcellular fraction. Figure 4A shows a significantly enhanced expression in differentiated cells at 7 days with respect to controls. Regarding vinculin recruitment, results showed a significant increase in membrane fraction at 7 days of differentiation and, consequently, a reduction in the cytoplasm fraction, although not significant (Figure 4B,C).

**Figure 5:**
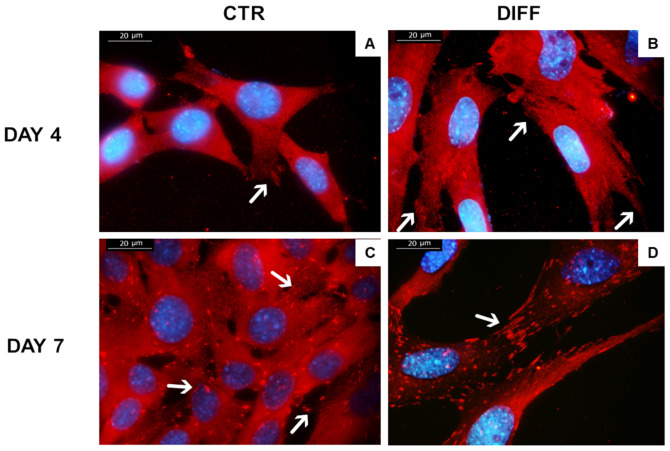
H9c2 cell morphology obtained by vinculin (red) staining. Images represent control cells at 4 (**A**) and 7 (**C**) days and differentiated cells at 4 (**B**) and 7 (**D**) days. Images are representative of all of the results obtained in the different experimental conditions. White arrow indicates vinculin in focal adhesions. Scale bar: 20 µm.

Morphological changes (length) and number of FAs per cell were evaluated after 7 days of differentiation (Figure 5). Cells showed a typical cardiomyocyte-like morphology of adherent cells with focal adhesion spindle-shape characteristics. Moreover, the signal of vinculin in FAs was shown to be more evident with time and differentiation, confirming the Western blot results.

**Figure 6:**
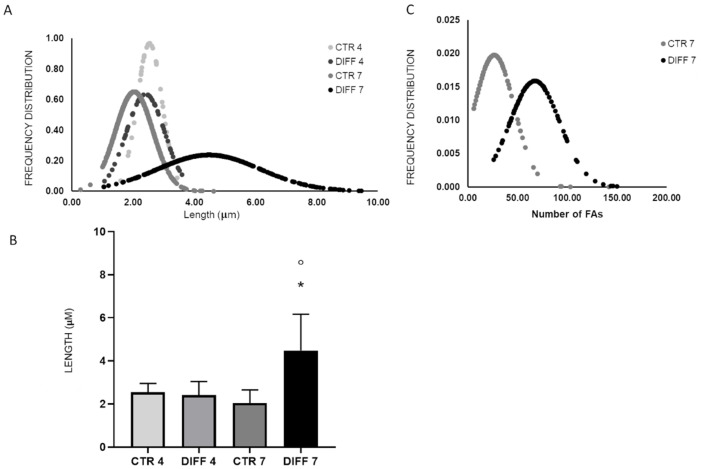
Morphometric analysis length (**A**,**B**) and number per cell (**C**) of H9c2 focal adhesions grown for 4 and 7 days in control and differentiation medium. Data are expressed as Gaussian curves with n ≥ 100. Measures were taken from representative fluorescence microscopy images (100× magnification). Statistical significance with respect to the relative controls (* *p* ≤ 0.05); statistical significance with respect to differentiated cells at day 4 (° *p* ≤ 0.05).

Remarkably, morphometric analysis revealed that the length of FAs increased significantly at 7 days of differentiation compared to the relative control and to cells differentiated for 4 days (Figure 6A,B). Furthermore, a higher number of FAs per cell was detected after 7 days of differentiation compared to the relative control (Figure 6C).

### 2.3. Activation of Focal Adhesions upon Mechanical Stress

**Figure 7:**
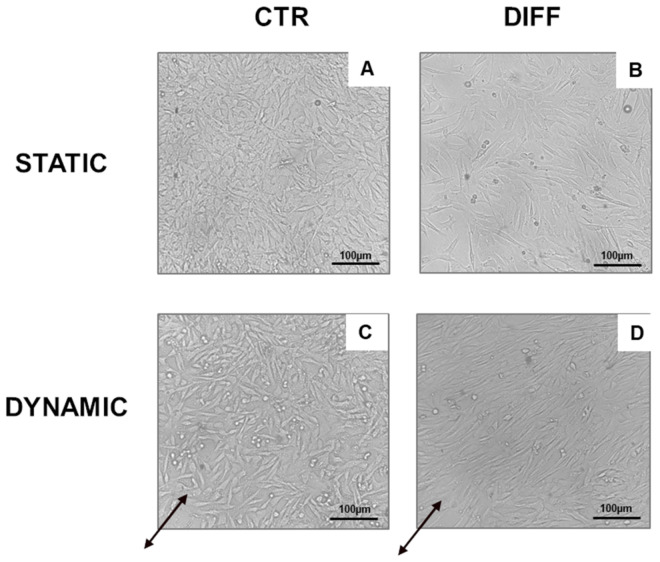
Morphological characteristics of H9c2 cardiomyocytes maintained under static (**A**,**B**) and dynamic (5% substrate deformation, 1 Hz frequency) conditions (**C**,**D**) for 7 days. Black arrows indicate the stretching direction. Images are representative of the results obtained in the different experimental conditions. Scale bar: 100 µm.

To highlight the importance of FAs in cardiac phenotype, H9c2 cardiomyocytes were subjected to a cyclic mechanical stress of 5% elongation and 1 Hz of frequency for 4 and 7 days in growth and differentiation medium. Figure 7 shows morphological characteristics of H9c2 grown for 7 days in static and dynamic conditions in growth and differentiation medium. After mechanical stretching, cells were oriented and aligned in parallel to the tension direction. The stretch direction is shown in Figure 7 with black arrows. These data suggest that mechanical stretch translates orientation signals in the cardiomyocytes.

**Figure 8:**
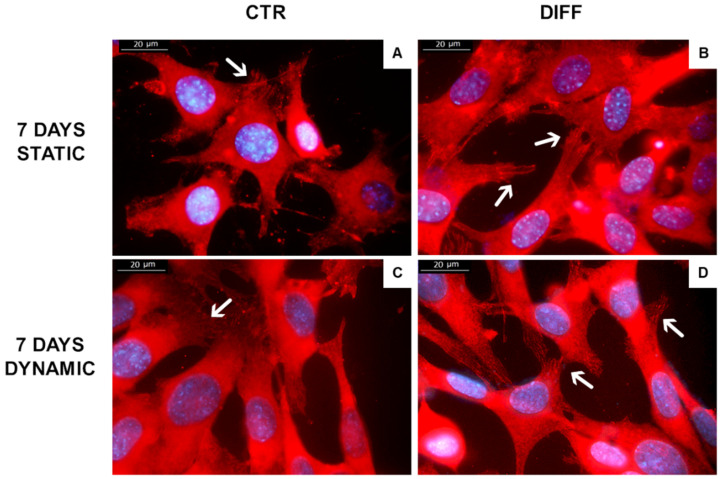
Expression and distribution of vinculin (red) staining in H9c2 cells cultured in static (**A**,**B**) and dynamic (**C**,**D**) conditions (5% substrate deformation, 1 Hz frequency). The experiments were repeated three times. Representative immunofluorescence micrographs are shown. White arrow indicate vinculin in focal adhesions. Scale bar: 20 µm.

Moreover, we assessed the expression of FAs during H9c2 differentiation, measuring the fraction of H9c2 cells positive for vinculin by immunofluorescence. Samples subjected to mechanical stress showed a strong presence of vinculin signals in the membrane of differentiated and mechanically stressed cells, in contrast to control cells where the presence of vinculin was mainly related to the cytoplasm (Figure 8).

**Figure 9:**
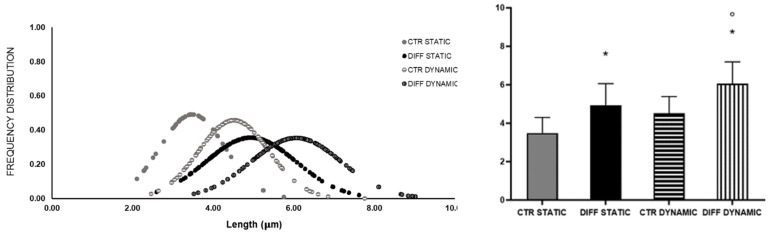
Morphometric analysis (length) of H9c2 focal adhesions grown for 7 days in growth and differentiation medium under static and dynamic (5% substrate deformation, 1 Hz frequency) conditions. Data are expressed as Gaussian curves with n ≥ 100. Measures were taken from representative fluorescence microscopy images (100× magnification). Statistical significance with respect to the relative controls (* *p* ≤ 0.05); statistical significance with respect to differentiated cells at day 4 (° *p* ≤ 0.05).

Intriguingly, morphometric analysis shown in Figure 9 revealed a significant shift of the Gaussian curve representing the length of FAs formed in differentiated cells at 7 days. These data confirm the proportional correlation between the morphological reorganization of FAs and mechanical stress.

## 3. Discussion

Cardiac differentiation is a dynamic process involving several biochemical pathways, regulated by internal and external stimuli, and sensor molecules. Among them, costameres and focal adhesions (FAs), the two main structures of cell–cell and cell-matrix (ECM) adhesion, have proved to play a central role in cardiomyocyte differentiation [10]. Within the numerous FA proteins that link the cytoplasmic domains of integrin with F-actin filaments, vinculin plays a pivotal role in regulating FA dynamics [9,10,19].

Cardiomyoblasts induced to differentiate showed a clear enhancement of GATA4 and Mef2c genes after 4 days of differentiation, indicating an early response to cardiac phenotype. These two early genes are highly expressed at the beginning of differentiation and can interact with several transcription factors, activating a cascade of downstream delayed genes, such as cTnT [40]. In particular, it is known that Mef2c is not only regulated during early development but also plays a decisive role when somatic stem cells differentiate into cardiac myocytes [41,42]. On the other hand, previous works report that GATA4 is expressed at high levels in neonatal myocytes and less in adult cardiac myocytes [41]. Upregulation of the cardiac progenitor gene NKX 2.5 was also observed to be significant at day 7. Moreover, results revealed that, after 7 days of differentiation, the expression of cardiac sarcomeric protein cTnT was upregulated, indicating an effective differentiation towards mature cardiomyocytes. In agreement with our results, Ruiz and colleagues showed that gene expression of cTnT was significantly increased at days 6–7 in H9c2 differentiated with RA compared to non-differentiated cells [43].

An important effect on the regulation of actin cytoskeleton and, thus, on cardiac differentiation, occurs at the integrin level. As mentioned before, the activation of integrins through ligand binding causes their subsequent clustering, followed by the activation of downstream cytoskeletal signaling proteins [35,44,45,46,47]. Among them, focal adhesion kinase (FAK) has been shown to play a critical role in cardiomyogenesis [22,23,24]. In particular, the FAK–Src complex activates downstream signaling pathways, leading to activation of phosphoinositide 3-kinase, protein kinase C, ERK, Jun N-terminal kinase, and p38 mitogen-activated protein kinase [48,49]. Torsoni and colleagues demonstrated the relevant role of FAK auto-phosphorylation at Tyr-397 on the recruitment of Src family kinases and costamere formation [50].

Moreover, the recruitment and activation of additional cytoplasmic linker proteins, such as vinculin, are also responsible for integrin clustering and, consequently, FA formation [51]. The interaction of vinculin with integrin receptors, through talin involvement, is considered the major link between FAs and the actin network having a direct effect on cytoskeleton dynamism. Additionally, the confinement of vinculin in FAs determines the recruitment of paxillin, which, in turn, promotes the recruitment and interaction of other proteins [9,13,14]. The contribution of vinculin in FA formation was also confirmed by the observation that the open (active) conformation is mainly recruited in the focal adhesion site; meanwhile, the inactive form is mainly present in the cytoplasm, thus suggesting vinculin redistribution during focal adhesion assembly [2,13,52]. Based on these findings, our data show that differentiation induces vinculin translocation, leading to strengthening of the integrin-based ECM adhesion complex.

The role of vinculin in FA formation and cell maturation was also confirmed by morphometrical analysis; in fact, differentiated cells exhibited higher number and length values of vinculin-positive focal contacts.

Additionally, it is known that mechanical stimuli are directly correlated to cell migration, proliferation, maturation, differentiation, organ preservation and ultimately disease programming [53]. In particular, physical cues play a key role from the earliest stage of development by contributing to tissue morphogenesis and homeostasis, especially in mechanically active tissues, such as myocardium [3,25,26,33]. In this regard, several in vitro models have been engineered to study the impact of mechanical signals on cell and tissue behavior [54,55,56]. Here, we demonstrated that a cyclic mechanical stress (5% substrate deformation, 1 Hz frequency) can promote cardiomyocyte alignment and vinculin activation. From a morphological point of view, we observed that cells subjected to mechanical stretch appear more spindled and elongated than the static group, characterized by a flat shape. Moreover, cells showed the ability to parallel orient with respect to the applied force. This observation is in good agreement with the statement that when a tensile force is applied to adhesive cells, the actin stress fibers are formed and oriented parallel to the direction of stretch [27,35,38]. This actin rearrangement was confirmed, since when a stretch was applied, the helical structure of actin filaments changed, reducing their affinity with cofilin and increasing their association with myosin II, leading to assembly and stabilization of the actin “stress” fibers [37,57].

We also found that mechanical stretch has a significant effect on force-sensitive proteins. In particular, vinculin morphology changed, resulting in modified length. These results agree with previous works that reported that sustained stress conditions activate focal contact molecules, leading to the activation of downstream regulators, including kinases, phosphatases, and transcriptional modulators, which stimulate different gene programs required for hearth remodeling [26,27,58]. Specifically, several studies outlined that mechanical stress has an important effect on the vinculin force-sensitive factor, controlling its activation and localization [59,60]. For example, Golji et al. (2011) predicted, from their computational simulation, that vinculin in maturating nascent adhesions was able to facilitate the F-actin linkage when a mechanical stress was present [61]. Other studies found that the activation state of vinculin is itself regulated by mechanical stretch, resulting in a more extensive binding of the protein with F-actin filaments. For example, Carisey et al. found that when mechanical stimuli decrease, FAs start to become labile and disassemble due to the insufficient tension necessary to keep vinculin in an active conformation [38]. All of these findings were also confirmed by in vivo studies performed on zebrafish models in which the mechanical forces generated, in this case by the cardiac contractility, were able to regulate the F-actin rearrangement, thus allowing cardiomyocyte myofilament maturation through the vinculin (VCL)–SSH1–CFL axis [60].

## 4. Materials and Methods

### 4.1. Cell Culture

Rat embryonic cardiomyoblasts (H9c2) (ATCC CRL-1446, Mannassas, VA, USA) were maintained at 37 °C in humidified atmosphere (5% CO_2_) and cultured in growth medium composed by Dulbecco’s Modified Eagle Medium (DMEM) (Euroclone, Milano, Italy) supplemented with 10% (*v*/*v*) fetal bovine serum (FBS), penicillin (100 U/mL), streptomycin (0.1 mg/mL), amphotericin (0.25 µg/mL) and L-Glutamine (2 mM) (all products from Euroclone, Milano, Italy). Cells were passed after 70–80% confluence was achieved. The differentiation of embryonic cardiomyoblasts into a mature phenotype was induced 24 h after seeding, reducing the percentage of serum (1% FBS) followed by the addition of 1 µM retinoic acid (RA) (Sigma, Milano, Italy). The stimulus (1% FBS + RA) was renewed every 2 days.

### 4.2. Total RNA Isolation and Real-Time Quantitative PCR (qRT-PCR)

H9c2 cells were seeded at a density of 1 × 10^3^ cells/cm^2^ and allowed to adhere for 24 h. Cells were then cultured for 4 and 7 days in growth (10% FBS, CTR) and differentiation (1% FBS + RA, DIFF) media.

*Total RNA extraction*. Collection of total RNA extraction was performed at 4 and 7 days using TRIzol (Thermo Fisher Scientific, Milano, Italy). Samples were collected and stored at –80 °C until RNA purification, performed according to the manufacturer’s protocol (Sigma Aldrich, Milano, Italy). The concentration and the purity of the extracted RNA were determined through the absorbance reading at 260 and 280 nm, using Nanodrop (Thermo Fisher Scientific, Milano, Italy). Afterward, DNAse treatment (DNAse I, Fermentas, St. Leon-Rot, Germany) was performed in order to digest single- and double-stranded DNA to oligodeoxy-ribonucleotides containing a 5′-phosphate.

*RNA retro-transcription*. cDNA was synthetized from 1 µg of extracted RNA according to RevertAid™ H Minus First Strand cDNA Synthesis Kit (Thermo Fisher Scientific, Milano, Italy) using the oligo (dT) primers. cDNA obtained was stored at −20°C until further use.

*Real-time quantitative PCR*. qRT-PCR was performed in 20 μL reaction volume containing 1 μL of RT products, 10 μL Sso-Fast EVA Green SMX (BioRad, Hercules, CA, USA) and 500 nM each forward and reverse primers, as indicated in Table 1. Strips were placed in CFX Connect Thermal Cycler (BioRad, Hercules, CA, USA) with a reaction cycle of 95 °C for 1 min, followed by 45 cycles of 98 °C for 5 s and anneal–extend step for 5 s at 60 °C. Results were exported in CFX Maestro Software (BioRad, Hercules, CA, USA) and analyzed in Excel (Microsoft, Redmond, WA, USA). All experiments were performed in triplicate.

### 4.3. Western Blot Analysis

Western blot was performed on cells seeded at a density of 1 × 10^3^ cells/cm^2^ and cultured in growth and differentiated medium for 4 and 7 days.

*Total lysis*. After 4 and 7 days of culture, H9c2 cells were lysed in boiling SDS (Tris-HCl 1 M pH 7,4, SDS 10%, PBS pH 7.4, ultrapure water), and cellular lysates were collected and stored at –20 °C. Protein concentration was determined using bicinchoninic acid assay (Pierce, Thermo Scientific, Milan, Italy). Samples were prepared for electrophoresis by dissolving in Laemmli Sample Buffer (62.5 mM Tris-HCl pH 6.8, 10% glycerol, 5% beta-mercaptoethanol, 0,005% bromophenol blue, 2% SDS) (Sigma Aldrich, Milano, Italy). Electrophoresis was performed using Sodium Dodecyl Sulphate-PolyAcrylamide Gel (SDS-PAGE) using 7.5% N, N’-methylenebisacrylamide (acrylamide) and then electrophoretically transferred to a nitrocellulose membrane (Amersham Biosciences, Little Chalfont, UK). Blotted proteins were blocked with 5% non-fat dried milk on PBS for 1 h at room temperature, then incubated overnight at 4 °C with mouse anti-vinculin (Merck Millipore, Milano, Italy), mouse anti-alpha-tubulin (Merck, Milano, Italy) and mouse anti-cardiac troponin T (cTnT) (Abcam, Milano, Italy) primary antibodies at a ratio of 1:1000. After washing, membranes were incubated with HRP-conjugated secondary antibody (1:2000; Perkin-Elmer, Milan, Italy) for 1 h at room temperature, and bands were visualized using a chemosensitive visualizer (ChemiDocTM Touch Imaging System, Bio-Rad, Milano, Italy). Densitometric analysis was performed using ImageLab (BioRad, Hercules, CA, USA) software. Tests were repeated three times.

*Subcellular fraction*. At the end of each time point, cells were lysed in subcellular fractionation buffer (10 mM Tris-HCl, pH 8, 1.5 mM MgCl2, 5 mM KCl, 50 μg/mL leupeptin, 5 μg/mL pepstatin A, 2 mM phenylmethylsulfonylfluoride), for 20 min on ice. Samples were mechanically disrupted with a manual Potter homogenizer (VWR, Milano, Italy) and centrifuged at 25,000× *g* for 45 min. The soluble cytoplasmic and insoluble membrane fractions were then recovered and analyzed directly by Western blot after SDS-PAGE separation as previously described. Tests were performed in triplicate.

### 4.4. Densitometry

A semiquantitative evaluation was performed on results obtained from Western blot analyses. Briefly, images acquired were analyzed with ImageLab (BioRad, Hercules, CA, USA) software (v3.0). Results were expressed as percentage ± standard deviation with respect to control at 4 days.

### 4.5. Mechanical Stimulation

To evaluate the role of mechanical stretch on FA and cardiomyocyte differentiation, rectangular silicone sheets (2.5 × 2 cm, 0.254 mm thick) made of polydimethylsiloxane (Specialty Manufacturing Inc., Saginaw, MI, USA) were used as deformable substrate. Silicone sheets were autoclaved (20 min at 121 °C) and coated with collagen type I tendons extracted from rat tails (50 µg/mL) for 1 h at 4 °C before cell seeding.

TC-3 mechanical stimulation bioreactor (Ebers Medical, Zaragoza, Spain) was used to apply a controlled mechanical stimulation in long-term culture using a specific and controlled stimulation profile, in terms of elongation, frequency and timing. The device is composed of an electronic control console, a loading frame, and a drive system that induces tension on the samples placed in the loading frame, until a predefined distance is reached, and then returns to the starting position. For the mechanical stimulation, H9c2 cells were seeded on coated silicon sheets, previously sterilized and positioned in the culture chambers, and maintained at 37 °C with 5% CO_2_ in a closed bath. In this system, control samples (CTR, H9c2 maintained in basic growth medium) and cardiac cells cultured in differentiating medium (DIFF) were subjected to a cyclic substrate deformation (5%) at a frequency of 1 Hz for 4 and 7 days. Static conditions were used as control.

### 4.6. Cell Morphology

Cell morphology (cell elongation, cell circularity and alignment) was observed comparing controls with mechanically stressed culture. An IM-3 OPTIKA inverted microscope equipped with a digital microscope camera (Leica DFC320, Wetzlar, Germany) was used.

### 4.7. Immunofluorescence Analysis

Cells were seeded at a density of 1 x 10^3^ cells/cm^2^ on both pre-coated (50 µg/mL collagen type I) glass coverslips and silicon sheets. After 4 and 7 days, H9c2 cultures were fixed with 4% formalin in PBS, permeabilized with 0.1% TritonX100 (Sigma Aldrich, Milano, Italy) and saturated with 5% Goat Serum (EuroClone, Milano, Italy) in PBS. Samples were then incubated overnight at 4°C with primary antibodies anti-vinculin (Sigma Aldrich, Milano, Italy), FAK and phospho-FAK Tyr397 (Invitrogen, Thermo Fisher Scientific, Milano, Italy) respectively diluted at 1:100, 1:250, and 1:500 in 2% Goat Serum (Euroclone, Milano, Italy) and 0.1% Triton X-100 (Sigma Aldrich, Milano, Italy) in PBS. Vinculin, FAK and phospho-FAK (Tyr397) were revealed by secondary Texas Red-labeled anti-mouse IgG antibodies (1:500; Vector TI-2000, Vector Laboratories, Newark, CA, USA). Cell nuclei were counterstained with 300 nM Diamidine-20-phenylindole dihydrochloride (DAPI, Sigma Aldrich, Milano, Italy). Mounting medium (60% glycerol in PBS) was finally used for optimizing fluorescence observation. To evaluate the formation of FAs through vinculin, FAK and pFAK expression, representative images were taken with the Leica DM2500 fluorescence microscope (Wetzlar, Germany) and acquired via Leica LAX acquisition software. A semi-quantitative analysis was performed using ImageJ software 1.52r (ImageJ, Bethesda, MD, USA), by counting the number of actin-related FAK-positive signals in 5 pictures for each condition. Experiments were performed in triplicate.

### 4.8. Morphometric Analysis

For analysis of FAs, the morphology of immunostained vinculin was evaluated by measuring the length and the total number of FAs per cell. In particular, the length of individual FAs was measured from images taken at 100× magnification by drawing the longitudinal axis of at least n > 100 vinculin positive signals, collected in 15 different microscope fields (DM2500 Leica microscope, Leica Microsystems, Milan, Italy). The total number of cell focal adhesions was measured on all cells in 10 representative fields collected at 20× magnification (DM2500 Leica microscope). Morphometric analyses were performed in triplicate. All data were analyzed using ImageJ software 1.52r (ImageJ, USA). The frequency distribution of the collected values (length and total number of FAs) was reported as a Gaussian function.

### 4.9. Statistical Analysis

All data were expressed as mean values ± standard deviation. Student’s *t*-test and two-way ANOVA with Bonferroni’s multiple comparison test were used for evaluating the significance of the results obtained. p-value was calculated and the differences between variables with a value of *p* < 0.05 were considered statistically significant (* *p* < 0.05). Each experiment was repeated three times.

## 5. Conclusions

Taken together, these findings established the importance of FAs as having a strong impact on cytoskeletal (F-actin) rearrangement and, consequently, on cardiomyocyte differentiation. Moreover, FAs have a role in transmitting mechanical cues required in heart functionality, outlining the positive effects of costameres and FAs in maintaining the cardiac contractility. Defects in FA assembly are linked to a variety of cardiac diseases, such as cardiomyopathies [10,60]. These findings provide a valuable insight into cardiovascular tissue engineering and regeneration, underlining the pivotal role of FAs during cardiomyocyte differentiation, thus suggesting their potential application as a regenerative target for improving cardiomyopathy outcomes. Finally, this work suggests that the application of a dynamic environment could be a valid strategy, by exploiting FAs, to obtain an improved differentiation. However, further investigations need to be performed to fully understand the link between mechanical stress, FA changes and cardiac differentiation.

## Figures and Tables

**Table 1:** Primers used for qRT-PCR. T.m.

*Gene*	*Forward Primers*	*Reverse Primers*	*T.m. ^a^*
β-actin	gatgacccagatcatgtttgaga	gtctccggagtccatcacaat	53 °C
gata-4	caactgccagactaccaccac	ccatggagcttcatgtagagg	52 °C
mef2c	tgctgtgcgactgtgagattg	cgtgcggctcgttgtactcg	59 °C
nkx2.5	tccgagcctggtagggaaa	aggtcaacaaaaggggatgga	55 °C

^*a*^ Temperature of melting.

## Data Availability

Not applicable.
